# Knowledge domain and emerging trends in diabetic cardiomyopathy: A scientometric review based on CiteSpace analysis

**DOI:** 10.3389/fcvm.2022.891428

**Published:** 2022-08-25

**Authors:** Shiyi Tao, Deshuang Yang, Lanxin Zhang, Lintong Yu, Zihan Wang, Lingling Li, Jin Zhang, Ruiqi Yao, Li Huang, Mingjing Shao

**Affiliations:** ^1^Graduate School, Beijing University of Chinese Medicine, Beijing, China; ^2^Oncology Department, Guang'anmen Hospital, China Academy of Chinese Medical Sciences, Beijing, China; ^3^Department of Integrative Cardiology, China-Japan Friendship Hospital, Beijing, China

**Keywords:** diabetic cardiomyopathy, scientometrics, bibliometrics, Web of Science, mapping knowledge domain, CiteSpace, visualization analysis

## Abstract

**Objective:**

To review the literature related to diabetic cardiomyopathy (DCM), and investigate research hotspots and development trends of this field in the relevant studies based on CiteSpace software of text mining and visualization in scientific literature.

**Methods:**

The relevant literature from the last 20 years was retrieved from the Web of Science (WoS) Core Collection database. After manual selection, each document record includes title, authors, year, organization, abstract, keywords, citation, descriptors, and identifiers. We imported the downloaded data into CiteSpace V (version 5.8.R2) to draw the knowledge map and conduct cooperative network analysis, cluster analysis, burst keyword analysis, and co-citation analysis.

**Results:**

After manual screening, there were 3,547 relevant pieces of literature published in the last 18 years (from 2004 to 2021), including 2,935 articles and reviews, which contained 15,533 references, and the number was increasing year by year. The publications of DCM were dedicated by 778 authors of 512 institutions in 116 countries. The People's Republic of China dominated this field (1,117), followed by the USA (768) and Canada (176). In general, most articles were published with a focus on “oxidative stress,” “heart failure,” “diabetic cardiomyopathy,” “dysfunction,” “cardiomyopathy,” “expression,” “heart,” “mechanism,” and “insulin resistance.” Then, 10 main clusters were generated with a modularity Q of 0.6442 and a weighted mean silhouette of 0.8325 by the log-likelihood ratio (LLR) algorithm, including #0 heart failure, #1 perfused heart, #2 metabolic disease, #3 protective effect, #4 diabetic patient, #5 cardiac fibrosis, #6 vascular complication, #7 mitochondrial dynamics, #8 sarcoplasmic reticulum, and #9 zinc supplementation. The top five references with the strongest citation bursts include “Boudina and Abel”, “Jia et al.”, “Fang et al.”, “Poornima et al.”, and “Aneja et al.”.

**Conclusion:**

The global field of DCM has expanded in the last 20 years. The People's Republic of China contributes the most. However, there is little cooperation among authors and institutions. Overall, this bibliometric study identified the hotspots in DCM research, including “stress state,” “energy metabolism,” “autophagy,” “apoptosis,” “inflammation,” “fibrosis,” “PPAR,” etc. Thus, further research focuses on these topics that may be more helpful to identify, prevent DCM and improve prophylaxis strategies to bring benefit to patients in the near future.

## Introduction

The number of people with diabetes is growing rapidly, and it is estimated that the number of adults with diabetes worldwide will reach 300 million by 2025 (1). It was found that 16.9% of diabetic patients suffered from diabetic cardiomyopathy (DCM), which shortened their life span and reduced the quality of life (2). Diabetic myocardial injury is a secondary injury to diabetic microangiopathy, proposed by Rubler in 1972, and manifests the clinical findings noted in cardiomyopathy, primarily cardiomegaly and congestive heart failure (3). Its pathologic lesion is characterized by diffuse myocardial fibrosis, cardiac hypertrophy, and diabetic microangiopathy (3). DCM is characterized by diastolic dysfunction in the early stage and systolic dysfunction in the late stage (4, 5). Both type 1 diabetes (insulin-deficient) and Type 2 diabetes (insulin-resistant) can progress to DCM. Type 2 diabetes accounts for 90–95% of DCM cases and is often associated with obesity (4). It was found that although the pathological manifestations of cardiomyopathy caused by type 1 and type 2 diabetes were not completely consistent, both of them could represent clinical phenotypes. The difference between the two types needs to be further studied (6).

DCM has been a concern by scholars since it was proposed in 1972, but the related research on DCM did not show explosive growth until 2004 (3, 7). Researchers have studied the pathogenesis of DCM from different perspectives, such as impaired myocardial insulin signaling, mitochondrial dysfunction, endoplasmic reticulum (ER) stress, impaired calcium homeostasis, abnormal coronary microcirculation, activation of the sympathetic nervous system (SNS), activation of the renin-angiotensin-aldosterone system (RAAS), and maladaptive immune responses (8–15). There is a lack of accurate diagnostic criteria for DCM. Diagnostic criteria are not inclusive diagnoses, but exclusionary diagnoses (16). Therefore, DCM is often ignored by physicians, resulting in delayed diagnosis and treatment. There are still no effective drugs and/or methods to treat DCM. Conventional hypoglycemic drugs and anti-heart failure drugs were used for symptomatic remission (5).

Bibliometrics is the subject of quantitative analysis, which is analyzed in time order and according to the changes of measurement objects such as the keywords, the number of articles, and authors (17–20). Thus, the quantitative results are used to evaluate the research hotspots in a certain research field and explore the next research direction. In this article, CiteSpace was used to collate the literature related to DCM in the Web of Science (WoS) Core Collection database. Citation analysis, hypothesis analysis, cluster analysis, and data visualization were used to analyze literature related to DCM from 2004 to 2021. A knowledge map was drawn and the research hotspots and development trends in this field were summarized. In this study, we explored the knowledge domain, quantitative research mode, and trend of DCM to help researchers obtain accurate and complete information on DCM's research areas.

## Methods

### Data collection

The data used for bibliometric analysis was collected in the WoS Core Collection of Thomson Reuters including SCI-Expanded, SSCI, A&HCl, CPCI-S, CPCI-SSH, ESCI, CCR-Expanded, and IC. The literature search was limited to the last 20 years. The topic search consisted of index words about DCM as follows: “diabetic cardiomyopathy,” “diabetic cardiomyopathies,” “cardiomyopathies, diabetic,” or “cardiomyopathy, diabetic.” This search resulted in 3,547 records, including 2,935 articles and reviews, which contained 15,533 references. The search records were exported to CiteSpace for further analysis (21). Studies were downloaded on December 20, 2021. Each document record included title, authors, year, organization, abstract, keywords, citation, descriptors, and identifiers. The research data involved in this study was obtained from an open-access public database, so there are no ethical issues involved in this study.

### Inclusion criteria

Inclusion criteria were: (1) Peer-reviewed published original articles on DCM, including basic and clinical research; (2) Reviews on DCM; (3) Articles published in the last 20 years; and (4) Articles retrieved from the WoS.

### Exclusion criteria

Exclusion criteria were: (1) Articles not officially published; (2) Conference abstracts and proceedings, corrigendum documents; and (3) Repeated publications.

### Quality assessment

English articles which met the inclusion criteria and were “articles” or “reviews” were included in the analysis. Through the method of reading the full text, the unqualified articles were proffered and the qualified articles were identified. The literature screening was conducted independently by two researchers. The discrepancies were resolved by discussion. Otherwise, a third researcher would be invited to work together to resolve the disagreement.

### Visualization analysis

CiteSpace V (version 5.8.R2) was used for visualization analysis in this study. In the analysis of co-occurrence or cooperation networks, we typically selected the top 50 articles from each time slice. The time slice was set as 1 year, and the time slicing was from 2004 to 2021. Pruning methods such as pathfinder, pruning sliced networks and pruning the merged network were used to simplify the network atlas. The included literature was analyzed by authors, institutions, and countries. The co-occurrence analysis, cluster analysis, timeline analysis, and burst term detection were conducted with keywords as nodes. The thickness of the line between nodes was positively correlated with the degree of association between two nodes. Betweenness centrality (BC) was defined by Freeman in 1977. The BC parameter is calculated by the following Equation (1) (22).


(1) $$\begin{array}{r} BC_{i}=\sum_{i\neq j\neq k}\frac{n_{st}^{i}}{g_{st}} \end{array}$$

In Equation (1), *g*_*st*_ represents the number of shortest paths between node *s* and node *t*, and $n_{st}^{i}$ is the number of those paths that pass-through node *i*. The importance of each node in the network can be partially evaluated by the indicator of BC. A node with a high BC (≥0.1) was defined as a turning point and marked with a purple circle (23), and the thickness was positively correlated with the BC. The clarity of keyword clustering results was judged by module value (*Q*-value) and average contour value (*S*-value). When *Q* > 0.3, the clustering structure is clear and significant. When *S* > 0.5, the clustering result is reasonable; when ≥0.7, the clustering has high efficiency and credibility (24).

## Results and discussion

### Publication years and journals

**Figure 1:**
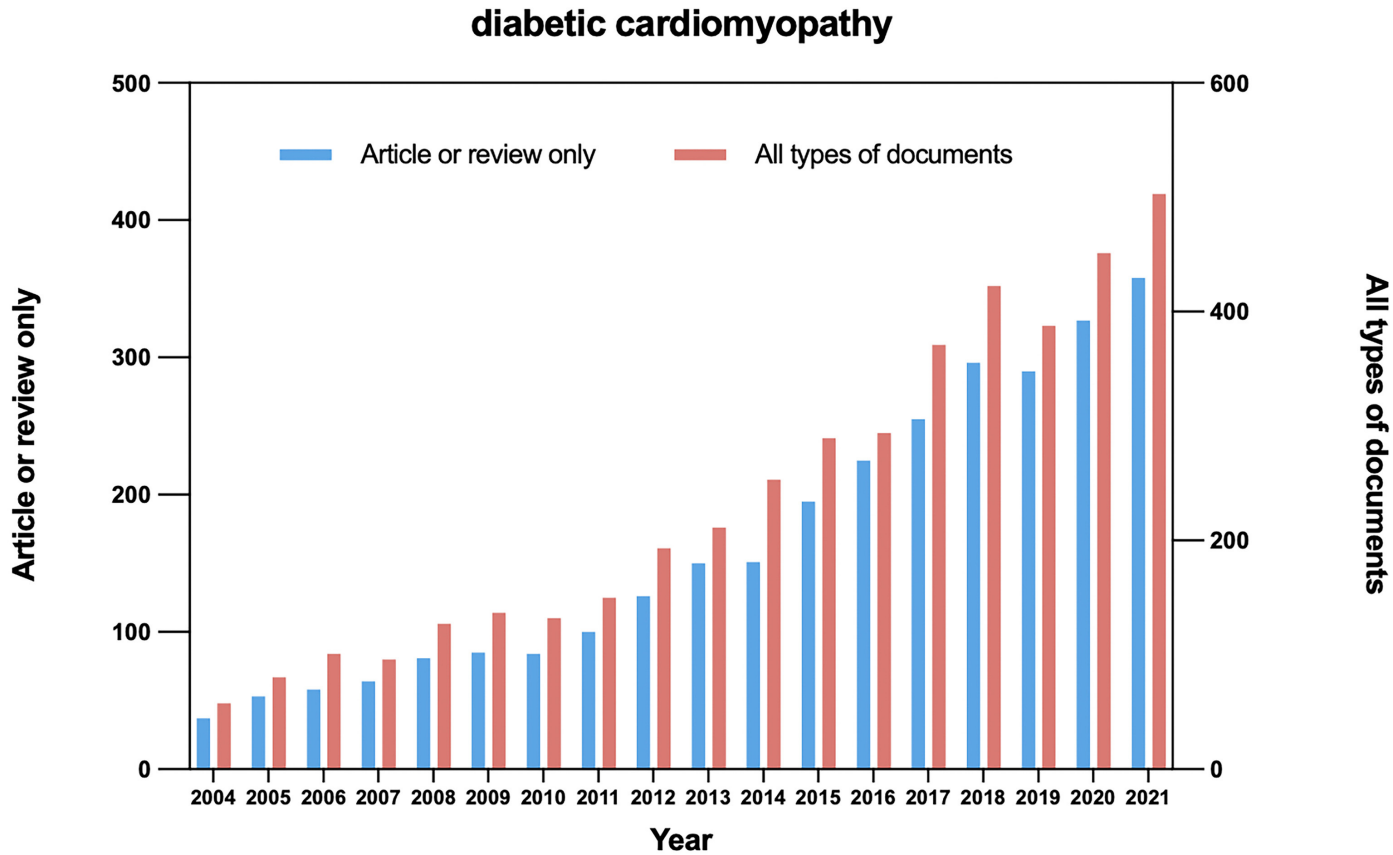
Time sequence of relevant papers of article/review and all types of documents on DCM published from 2004 to 2021 in WoS.

The first article about DCM that could be found in the WoS, “*In vivo* adenoviral transfer of sorcin reverses cardiac contractile abnormalities of diabetic cardiomyopathy,” was published in 2004 by Suarez et al. (7), which stood for the prototype of the DCM research field. Since that, with the persistent deepening of the research on DCM by investigators worldwide, the publications about DCM were growing continuously, but the total number of articles published each year was still low. The number of all types of published documents increased from 48 in 2004 to 419 in 2021, the number of published articles and reviews increased from 37 to 358, as shown in Figure 1. The correlation between the number of published papers and the published year series data indicates that the growth pattern in Figure 1 was very close to linear correlation.

All the articles and reviews recorded on DCM were distributed in 620 journals. Cardiovascular Diabetology ranked first in the number of publications (91), followed by the American Journal of Physiology-Heart and Circulatory Physiology (75), and Diabetes (54). The top 15 journals with the largest number of published DCM studies were listed in Table 1, which could provide important references and ideas for new investigators to further research.

**Table 1:** Top 15 most productive journals.

**Journals**	**Impact factor**	**The number of published papers**
Cardiovascular Diabetology	9.951	91
American Journal of Physiology-Heart and Circulatory Physiology	4.733	75
Diabetes	9.461	54
Journal of Molecular and Cellular Cardiology	5	53
Plos One	3.24	53
Frontiers in Physiology	4.566	44
Journal of Cellular and Molecular Medicine	5.31	44
Biochemical and Biophysical Research Communications	3.575	41
Oxidative Medicine and Cellular Longevity	6.543	41
Frontiers in Pharmacology	5.81	39
International Journal of Molecular Sciences	5.923	38
Scientific Reports	4.379	38
Molecular Medicine Reports	2.952	37
Biochimica et Biophysica Acta-Molecular Basis of Disease	5.187	36
Experimental and Therapeutic Medicine	2.447	34

### Co-authorship

**Figure 2:**
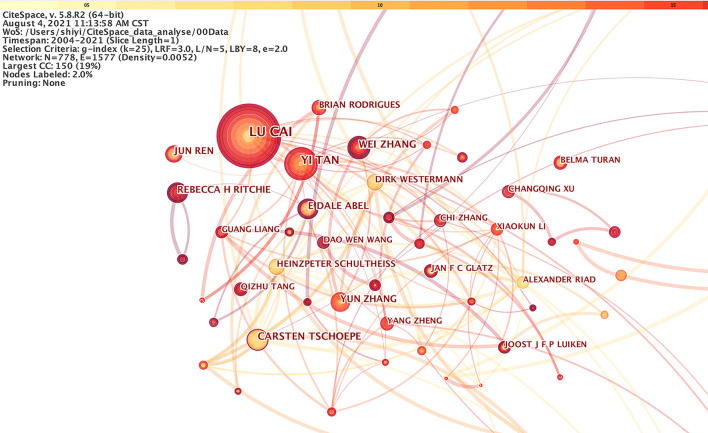
The cooperation network of productive authors.

Studies published from 2004 to 2021 were chosen with a time slice of 1 year for the analysis, and the selection criteria were the top 50% per slice. The co-authorship network was displayed in Figure 2. The size of the circle represented the number of studies published by the author. The color of circles stood for the authors in the same cluster. The shorter the distance between the two circles, the more cooperation between the two authors. The color of the circle represented the author of the same cluster. Yellow nodes represented earlier published studies, while red nodes represented more recently published studies. It could be noticed that many authors tend to collaborate with a relatively stable group of collaborators. Therefore, several major clusters of authors were generated, each of which usually had two or more core authors.

Figure 2 demonstrated that the most representative author in the field of DCM is LU CAI from the University of Louisville with a total of 56 published studies, followed by YI TAN from the University of Louisville. This analysis could offer highly personalized scientific research information about DCM for other investigators. LU CAI's team mainly explored the potential protection of metallothionein (MT) and nuclear respiratory factor-2 (Nrf2) from DCM (25–28). Zinc, curcumin, and sulforaphane (SFN) supplements may protect against DCM (29, 30). The team members were mainly from Louisville and Philadelphia in the United States, Ontario in Canada, Wenzhou, Huzhou, Shenyang, Jilin, and Beijing in China, and Al Ain in the United Arab Emirates. Their first article was picked up by WoS in 2004. Since then, a total of 311 articles, with 10,195 citations could be found in the WoS.

### Co-institute

**Figure 3:**
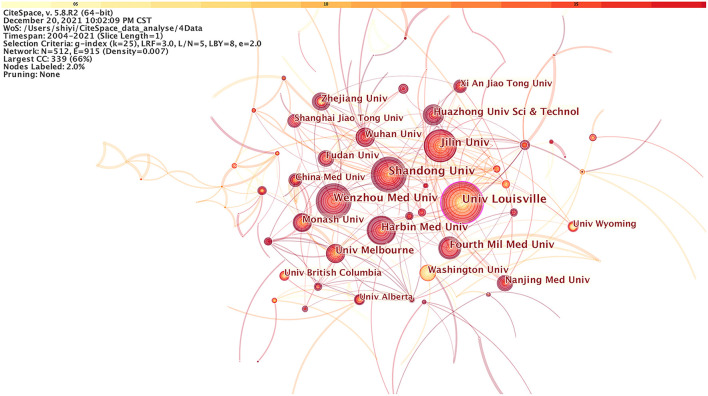
The cooperation network of productive institutes.

Two authors' institutes appear in the same article as one cooperation. CiteSpace software mainly judges cooperation based on the co-occurrence frequency matrix. Studies published from 2004 to 2021 were chosen with a time slice of 1 year for the analysis, and the top 50 most-cited or -occurring items were chosen from each slice. Figure 3 exhibited co-institutes in the field of DCM. Nodes represent institutes, and the size of each node corresponds to the co-occurrence frequency of the institutes. The size of the circle represents the number of papers published by the institute. The shorter the distance between two circles, the greater the cooperation between the two institutes. Purple rings indicated that these institutes have greater BC (no <0.1).

In addition, the publications of DCM were dedicated by 512 institutions, and there were 915 links in total between these nodes. As shown in Figure 3, the University of Louisville in the USA had published the largest number of studies (82), followed by Shandong University in China (76), and Jilin University in China (73). The BC of nodes of the both University of Louisville and Wenzhou Medical University was 0.1, indicating that the two institutions cooperate closely with other institutions. Meanwhile, there was also a cooperative relationship among other organizations that published the latest literature. These organizations included Harbin Medical University (60), Fourth Military Medical University (50), The University of Melbourne (44), Huazhong University of Science and Technology (42), Wuhan University (40), Monash University (39), and University of Washington (37), etc.

### Co-country

**Figure 4:**
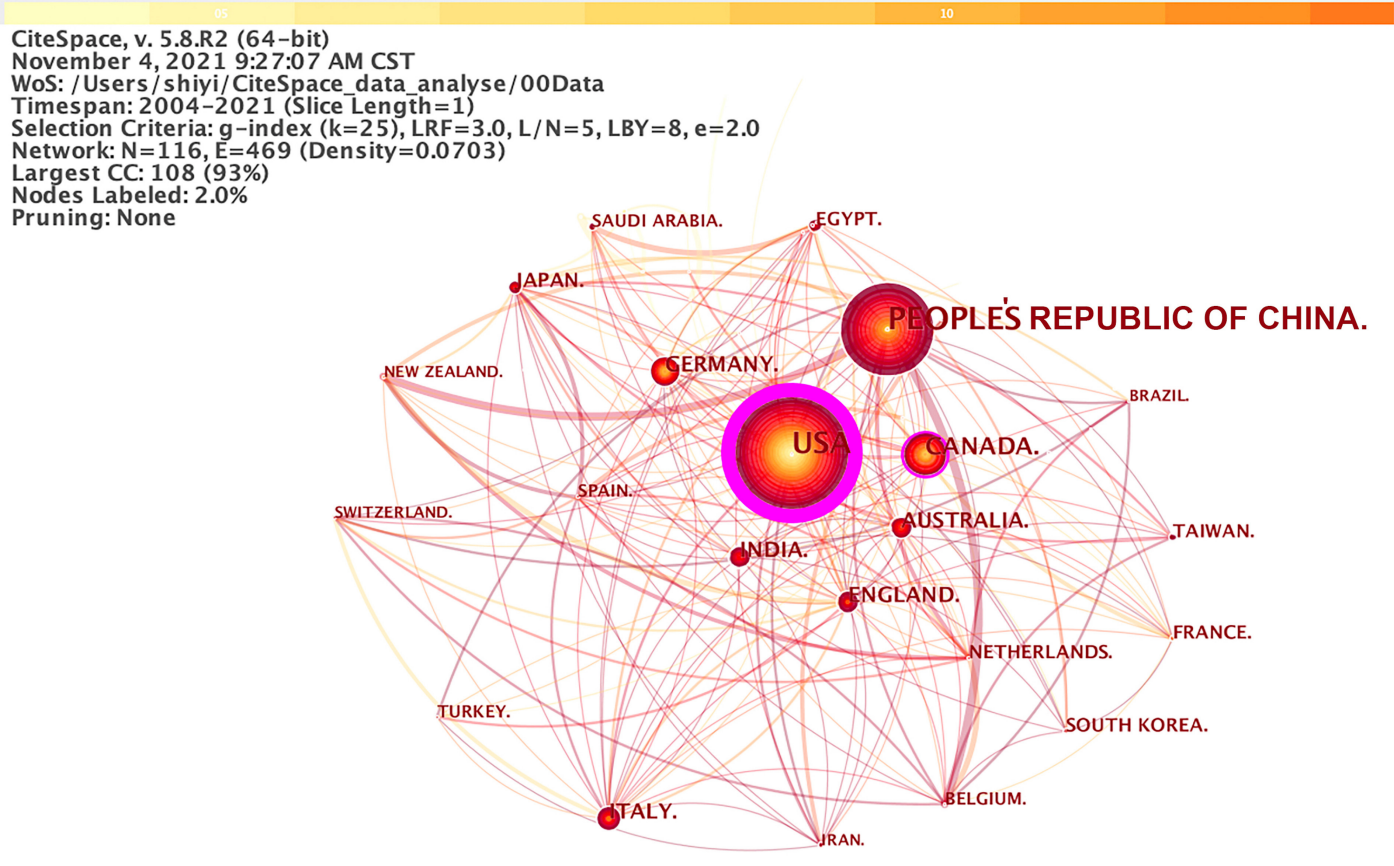
The cooperation network of productive countries.

Studies published from 2004 to 2021 were chosen with a time slice of 1 year for the analysis, and the top 50 most-cited or -occurring items were chosen from each slice. Figure 4 displayed co-country results in DCM research. The size of the circle represented the number of papers published by the country. The shorter the distance between the two circles, the greater the cooperation between the two countries.

According, there were 2,935 papers published in 116 countries. the People's Republic of China published the largest number of papers (1,117), followed by the United States (768), and Canada (176). Nevertheless, there were few nodes connected (only 469 links) compared with the cooperation at the institutional level. Among them, the node representing the USA had the highest BC (0.97), followed by Canada (0.13), Germany (0.13), and Italy (0.10). It shows that the United States had the most communication and cooperation with other countries.

### Co-occurring keywords analysis

**Figure 5:**
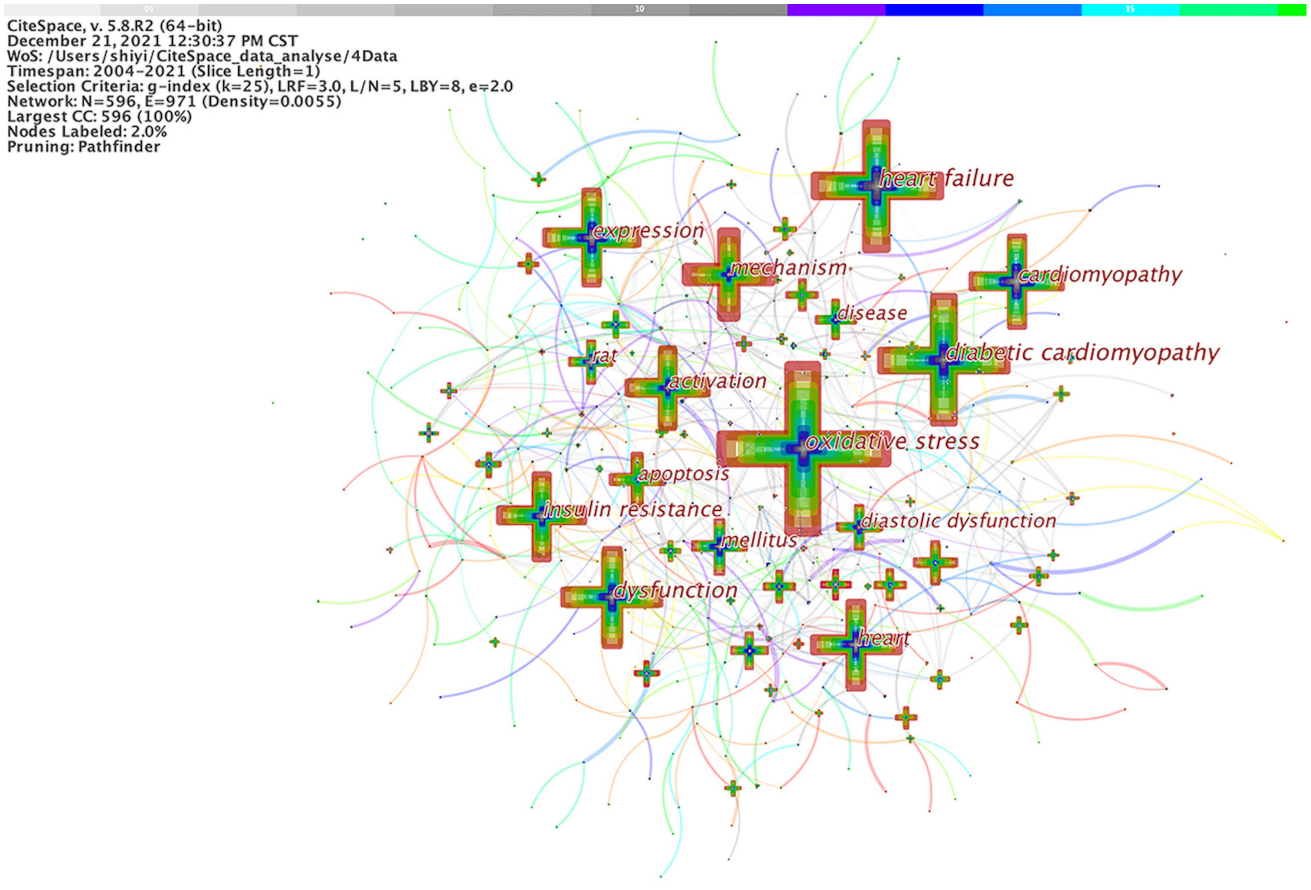
Analysis of co-occurring keywords in DCM research.

**Figure 6:**
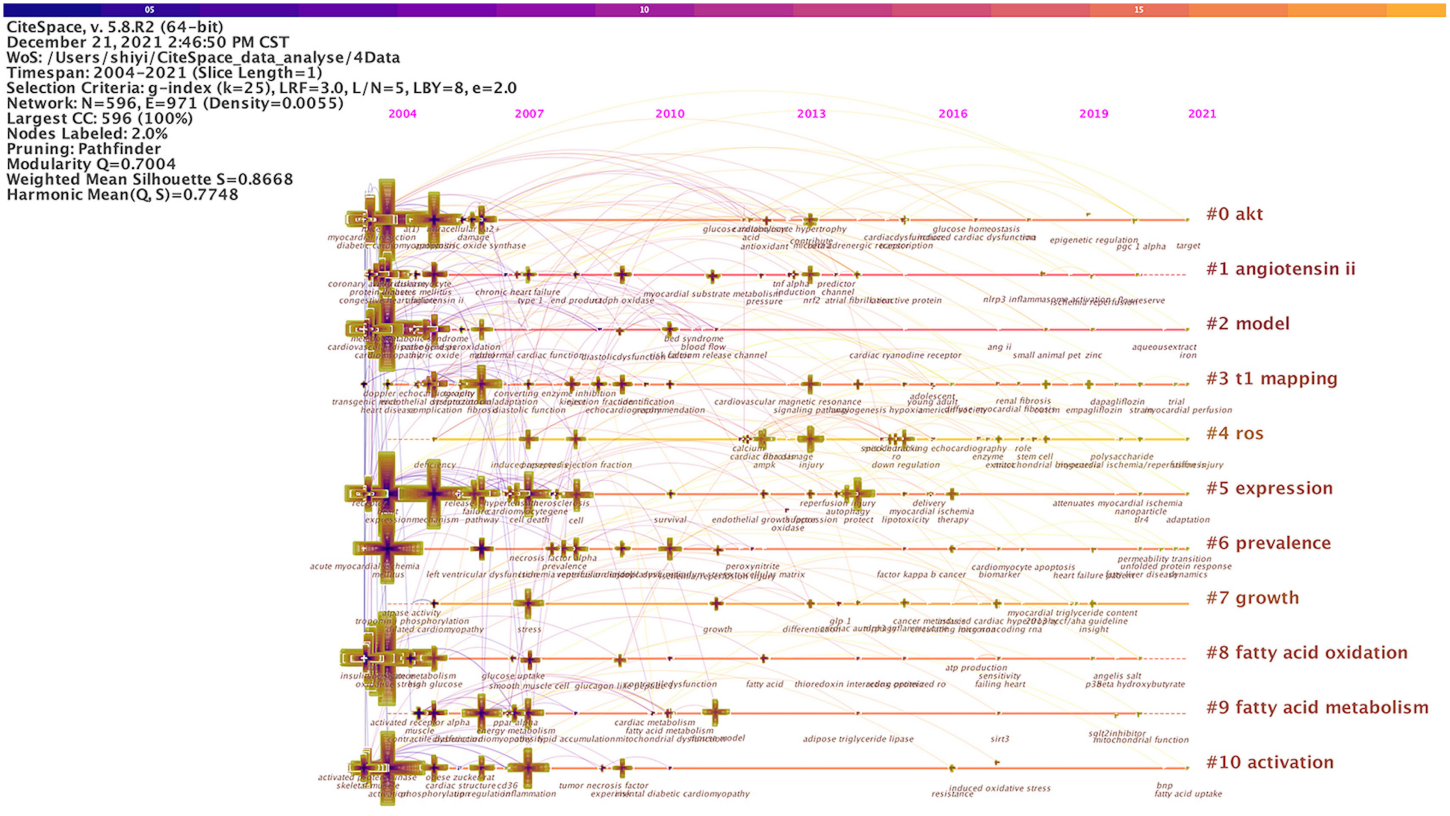
Recurring cardiomyopathy research after Figure 5 data are sorted into chronological order.

Co-occurring keywords reflect research hotspots in the field of DCM. Studies published from 2004 to 2021 were chosen with a time slice of 1 year for CiteSpace analysis. The top 50 most-cited or -occurring items were chosen from each slice. As shown in Figure 5, a simplified co-occurring keyword network was obtained with the pathfinder algorithm. A node represents a keyword, and the size of each node was consistent with the co-occurring frequencies of keywords. The color of the co-occurring links between the keywords represented the times when the keywords appear together, and the chronological order of colors were: oldest in gray, and newest in green. The maximum frequency was of “oxidative stress” at 663, followed by “heart failure” (524), “diabetic cardiomyopathy” (522), and “dysfunction” (413). Other commonly used words were “cardiomyopathy” (402), “expression” (393), “heart” (385), “mechanism” (375), “insulin resistance” (365), and so on. A few of these nodes marked with purple circles represent good BC and the importance of these keywords. In other words, these nodes represent emerging trends in the field of DCM, with the strongest bursts. Figure 5 was sorted by time zone to obtain Figure 6. Figure 6 shows the historical process of DCM research.

### Document co-citation analysis

**Figure 7:**
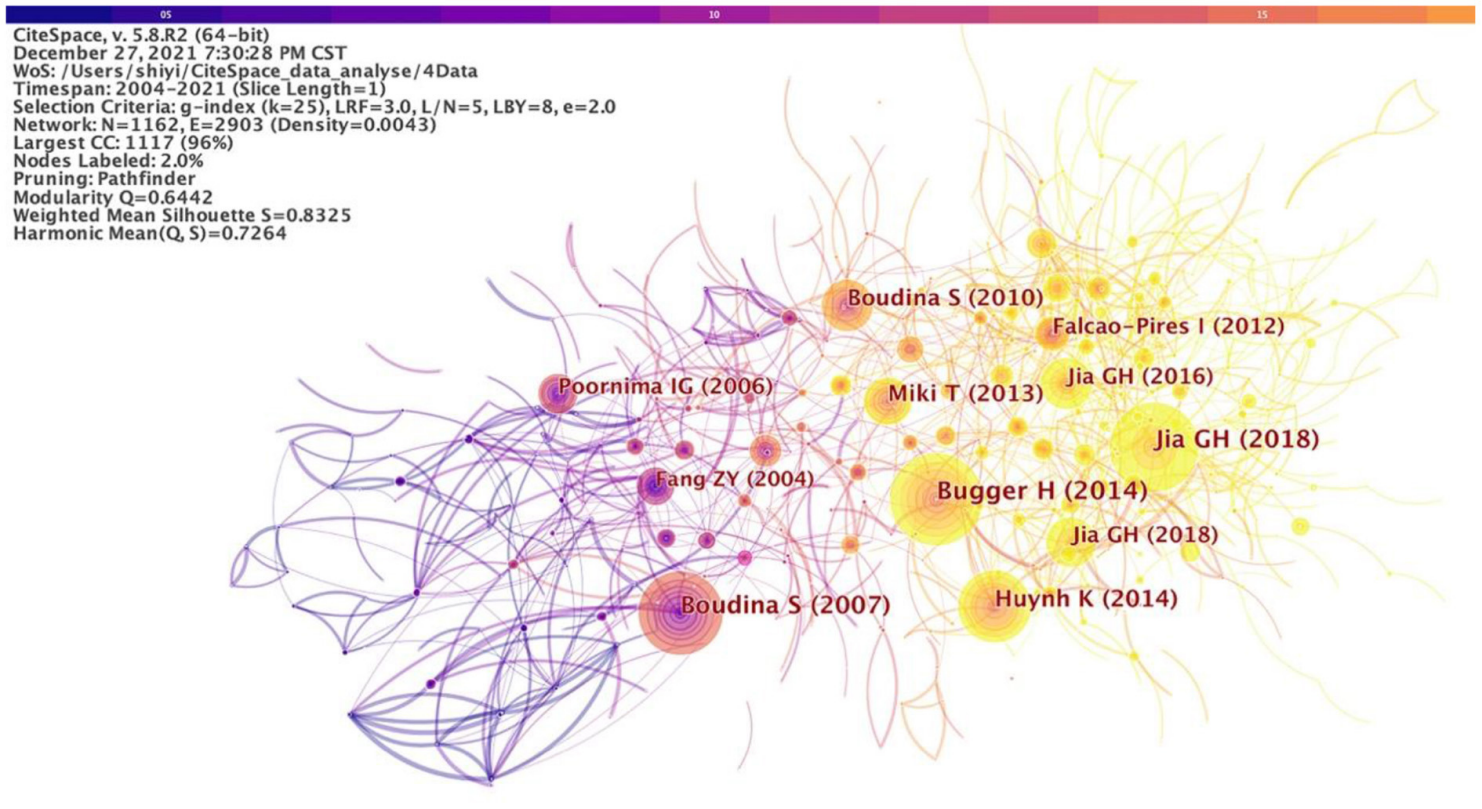
Document co-citation analysis in DCM research.

A total of 2,935 articles and reviews were analyzed and visualized through CiteSpace software, with a timespan from 2004 to 2021 and a time slice of 1. The selection criteria were the top 50 most cited or occurred items from each slice. The document co-citation network pruned by pathfinder was generated. A document co-citation network map was shown in Figure 7.

As a result, 1,162 unique nodes, 2,903 lines, and 10 main clusters were generated with a modularity Q of 0.6442 and a weighted mean silhouette of 0.8325. These nodes and links represent cited references and co-citation relationships, respectively. The more cited the study, the larger the node. The color and thickness of the circle in the node indicating the citation frequency at different periods. Line colors corresponded directly to the time slice. The cold colors represent earlier years, while warm ones represent more recent years. For example, purple lines represent studies co-cited in 2004, and yellow or orange lines visualized recent studies co-citation. The modularity Q and the weighted mean silhouette are two indicators to evaluate the clusters. *Q* > 0.3 means that the network is significant and the silhouette >0.5 means that the clustering result is rational. The citation year ring represents the citation history of this study. The color of the citation ring represents the corresponding citation time. The thickness of an annual ring is proportional to the number of citations in a time zone.

As shown in Table 2, the top-ranked item by citation counts was Bugger and Abel (15) in Cluster 0 with a citation count of 246, followed by Boudina et al. (31) in Cluster 8 with a citation count of 235, and Jia et al. (32) in Cluster 5 with a citation count of 231, Huynh et al. (33) in Cluster 0 with a citation count of 196, Boudina and Abel (34) in Cluster 6 with a citation count of 139, and Miki et al. (35) in Cluster 0 with a citation count of 138.

**Table 2:** Important diabetic cardiomyopathy references with high burst values.

**Citation counts**	**Sigma**	**Burst**	**Author**	**Year**	**BC**	**Source**	**Half-life**	**Cluster ID**
246	1.18	21.98	Bugger	2014	0.01	Diabetologia	4.5	0
235	2.39	86.07	Boudina	2007	0.01	Circulation	4.5	8
231	3.15	67.07	Jia	2018	0.02	Circ Res	1.5	5
196	1.1	18.4	Huynh	2014	0.01	Pharmacol Therapeut	3.5	0
139	1.66	33.15	Boudina	2010	0.02	Rev Endocr Metab Dis	5.5	6
138	2.14	16.46	Miki	2013	0.05	Heart Fail Rev	4.5	0
134	1.45	25	Jia	2016	0.01	Nat Rev Endocrinol	3.5	0
130	1.13	35.09	Jia	2018	0.00	Diabetologia	1.5	0
117	5.19	44.2	Poornima	2006	0.04	Circ Res	4.5	0
112	1.82	16.18	Falcao-Pires	2012	0.04	Heart Fail Rev	4.5	3
109	1.86	50.46	Fang	2004	0.01	Endocr Rev	5.5	4
98	1.86	36.02	Aneja	2008	0.02	Am J Med	4.5	4
85	1.14	10.4	Seferovic	2015	0.01	Eur Heart J	3.5	0
83	1.48	23.5	Zinman	2015	0.02	New Engl J Med	3.5	0
81	1.52	23.95	Giacco	2010	0.02	Circ Res	5.5	3

BC, betweenness centrality.

Bugger and Abel (15) was a review that mainly reviewed the molecular mechanisms of DCM. Boudina et al. (31) reported that mitochondrial uncoupling in the heart in obesity and diabetes is mediated by activation of uncoupling proteins (UCPs) independently of changes in expression levels. Jia et al. (32) summarized molecular mechanisms, structural and functional changes, and possible therapeutic approaches for the prevention and treatment of DCM. The pathophysiological processes of DCM include mitochondrial dysfunction, oxidative stress, increased formation, and deposition of advanced glycation end products (AGEs), impaired mitochondrial Ca2^+^ handling and function, inflammation, activation of RAAS and SNS, cardiac autonomic neuropathy, ER stress, microvascular dysfunction, and cardiac metabolic disorders. Pathways and proteins include AMP-activated protein kinase (AMPK), peroxisome proliferator-activated receptors (PPARs), sodium-glucose cotransporter 2 (SGLT2), protein kinase C (PKC), mitogen-activated protein kinase (MAPK), nuclear factor κB (NF-κB), Nrf2, microRNA (miRNA), and exosomes. Huynh et al. (33) considered the increased reactive oxygen species production and decreased antioxidant defense, as well as related protein signaling pathways and miRNA dysregulation in DCM. Boudina and Abel (34) illustrated the development of DCM by describing the structural, functional, and metabolic changes in the myocardium of DCM patients. Miki et al. (35) summarized the previous basic and clinical studies on DCM and point out the unsolved problems, that is, no clinical strategies have been established to prevent, treat, and improve the prognosis of DCM.

Research patterns and emerging trends in the knowledge system in terms of key clusters of articles were explored. A document co-citation analysis of DCM research produced 10 co-citation clusters. These clusters were labeled by indexed terms from their own citations. To characterize the nature of a cluster, noun phrases were extracted from the title of the article that cited the cluster by CiteSpace based on three specialized algorithms—log-likelihood ratio (LLR), latent semantic indexing (LSI), and mutual information (MI). Of the three, LLR generally gives the best results in terms of the uniqueness and coverage of cluster-related topics. The detailed information of the 10 clusters was summarized in Table 3. The values of the silhouette for each cluster were >0.7, indicating reliable and meaningful results. “Heart failure” was the largest cluster (#0) consisting of 146 members. The most active citer in this cluster was Bugger and Abel (15), “Molecular mechanisms of diabetic cardiomyopathy.” This paper reviewed the molecular mechanisms of DCM. Lip toxicity, mitochondrial dysfunction, ER stress, oxidative stress, increased AGEs signaling, and inflammation might promote fibrosis or apoptosis. The second-largest cluster (#1) in this knowledge domain, “perfused heart,” had 110 member articles, and the average year of publications was 2003. The most active citer to this cluster was An and Rodrigues (36), “Role of changes in cardiac metabolism in the development of diabetic cardiomyopathy.” This article focused on evaluating the acute and chronic regulation and dysregulation of cardiac metabolism in normal and insulin-resistant/diabetic hearts, and how these changes could contribute to the development of cardiomyopathy. The third-largest cluster (#2) was “metabolic disease” which had 104 members and the average year of publication was 2014. The most active citer to this cluster is Xie et al. (37), “Improvement of cardiac functions by chronic metformin treatment is associated with enhanced cardiac autophagy in diabetic OVE26 mice.” They found that decreased AMPK activity and reduced cardiac autophagy were vital events in the development of DCM through *in vivo* and *in vitro* experiments. Metformin-mediated chronic activation of AMPK prevented DCM by upregulating autophagy activity in DCM.

**Table 3:** The largest 10 clusters of diabetic cardiomyopathy research document co-citation, identified by subject headings.

**Cluster ID**	**Size**	**Silhouette**	**Mean (cite year)**	**Label (LLR)**	**Label (LSI)**	**Label (MI)**
0	146	0.755	2014	Heart failure	Diabetic cardiomyopathy	Mek inhibitor
1	110	0.842	2003	Perfused heart	Cardiac dysfunction	Mek inhibitor
2	104	0.880	2014	Metabolic disease	Diabetic heart	Mek inhibitor
3	99	0.787	2013	Protective effect	Protective effect	Mek inhibitor
4	95	0.791	2006	Diabetic patient	Diastolic dysfunction	Mek inhibitor
5	91	0.847	2016	Cardiac fibrosis	Cardiac fibrosis	Mek inhibitor
6	72	0.777	2013	Vascular complication	Vascular complication	Mek inhibitor
7	70	0.855	2010	Mitochondrial dynamics	Oxidative stress	Mek inhibitor
8	67	0.811	2003	Sarcoplasmic reticulum	Mouse model	Mek inhibitor
9	62	0.894	2003	Zinc supplementation	Zinc supplementation	Mek inhibitor

LSI, latent semantic indexing; LLR, log-likelihood ratio; MI, mutual information.

There were other clusters in Table 3 worth mentioning. For example, cluster #8 had the top-ranked burst article published by Boudina and Abel (38) among all clusters, with a burst of 86.07. This article represented the active area and emerging trends in the DCM field, such as impaired calcium homeostasis, upregulation of the RAAS, increased oxidative stress, altered substrate metabolism, and mitochondrial dysfunction. The second-ranked burst article was published by Jia et al. (32) with a burst of 67.07 in cluster #5. This work provided an update on the pathogenesis of DCM, summarized the latest research support for the mechanism, proposed exosome and circulating miRNAs as new potential research and treatment directions. The third-ranked burst article was published by Fang et al. (39) with a burst of 50.46 in cluster #4, which mainly summarized the clinical significance, pathogenesis, and therapeutic significance of DCM. The pathophysiology of DCM remained to be fully elucidated, and there was no clinical gold standard for diagnosis and treatment of the disease. In summary, the pathogenesis, gold standard diagnosis, and treatment were still problems that needed to be explored. Exosome and circulating miRNAs were emerging trends in DCM research.

### Emerging trends

A significant increase in research interests in the DCM field were highlighted by publications with citation bursts. Table 4 showed the top 25 references with the strongest citation bursts among a total of 1,162 references (1,162 unique nodes in Figure 7) from 2004 to 2021. Four of the top five studies were completed before 2010. The emerging trends have been discussed in detail above. Some representative studies emerged from different years among the 1,162 references. These articles gave expression to the emerging trends and reflected the development track of DCM, as listed in Table 5.

**Table 4:** The top 25 references with the strongest citation bursts.

**References**	**Year**	**Strength**	**Begin**	**End**	**2004–2021**
Boudina and Abel (38)	2007	86.07	2008	2015	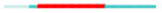
Jia et al. (32)	2018	67.07	2019	2021	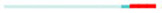
Fang et al. (39)	2004	50.46	2006	2012	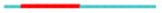
Poornima (40)	2006	44.2	2007	2014	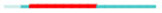
Aneja et al. (41)	2008	36.02	2010	2016	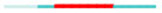
Jia et al. (8)	2018	35.09	2019	2021	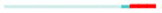
Boudina and Abel (34)	2010	33.15	2012	2018	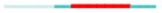
Cai et al. (28)	2006	28.13	2009	2014	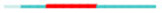
Hayat (42)	2004	27.19	2006	2012	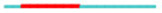
An and Rodrigues (36)	2006	25.1	2007	2013	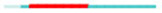
Jia et al. (14)	2016	25	2018	2021	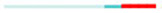
Giacco (43)	2010	23.95	2014	2018	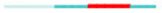
Asbun (44)	2006	23.62	2007	2014	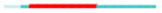
Zinman (45)	2015	23.5	2017	2021	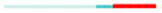
Finck et al. (46)	2002	23.31	2004	2010	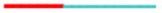
Bugger and Abel (15)	2014	21.98	2016	2021	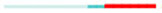
Westermann (47)	2007	21.27	2008	2015	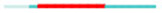
Asghar (48)	2009	19.67	2011	2014	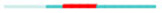
Taegtmeyer et al. (49)	2002	19.24	2004	2010	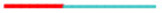
Finck et al. (50)	2003	19.24	2004	2011	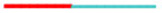
Frustaci et al. (51)	2000	18.92	2004	2008	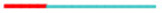
Poirier (52)	2001	18.84	2004	2009	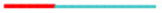
Trost (53)	2002	18.73	2004	2010	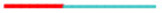
Huynh et al. (33)	2014	18.4	2015	2018	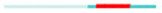
Liang (54)	2002	18.23	2004	2010	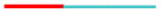

Blue line represents the base timeline, and red part indicates the burst duration of each reference.

**Table 5:** Representative references sorted by the beginning of the burst.

**References**	**Year**	**Strength**	**Begin**	**End**	**2004–2021**
Finck et al. (46)	2002	23.31	2004	2010	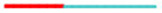
Taegtmeyer et al. (49)	2002	19.24	2004	2010	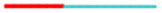
Finck et al. (50)	2003	19.24	2004	2011	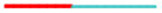
Frustaci et al. (51)	2000	18.92	2004	2008	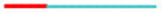
Poirier (52)	2001	18.84	2004	2009	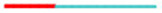
Trost (53)	2002	18.73	2004	2010	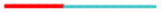
Liang (54)	2002	18.23	2004	2010	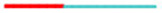
Fang et al. (39)	2004	50.46	2006	2012	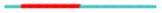
Hayat (42)	2004	27.19	2006	2012	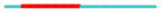
Poornima (40)	2006	44.2	2007	2014	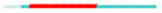
An and Rodrigues (36)	2006	25.1	2007	2013	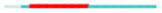
Asbun (44)	2006	23.62	2007	2014	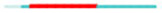
Boudina and Abel (38)	2007	86.07	2008	2015	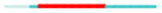
Westermann (47)	2007	21.27	2008	2015	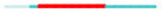
Cai et al. (28)	2006	28.13	2009	2014	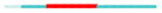
Aneja et al. (41)	2008	36.02	2010	2016	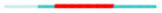
Asghar (48)	2009	19.67	2011	2014	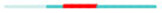
Boudina and Abel (34)	2010	33.15	2012	2018	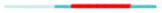
Giacco (43)	2010	23.95	2014	2018	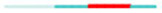
Huynh et al. (33)	2014	18.4	2015	2018	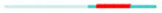
Bugger and Abel (15)	2014	21.98	2016	2021	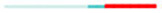
Zinman (45)	2015	23.5	2017	2021	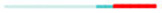
Jia et al. (14)	2016	25	2018	2021	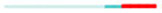
Jia et al. (8)	2018	67.07	2019	2021	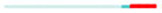
Jia et al. (32)	2018	35.09	2019	2021	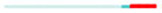

Blue line represents the base timeline, and red part indicates the burst duration of each reference.

The representative references reflecting the development history of DCM in terms of the beginning time of burst were displayed in Table 5. The earliest reference with the strongest citation bursts was published by Finck et al. (46). The burst duration of this article was from 2004 to 2010. It was one of the earliest reviews that talked about the important role of PPAR-α in the transcriptional control of cardiac energy metabolism. Subsequently, Taegtmeyer et al. (49) successfully demonstrated energy substrate metabolism and contractile function, the potential molecular mechanisms, and the gene expression of the DCM heart. The burst also last 7 years from 2004 to 2010 with a burst of 19.24. Finck et al. (50) tuned the PPAR-α mediated derangements by experiment on transgenic mice. This work provided a new link between dysregulation of the PPAR-α gene regulatory pathway and cardiac diabetic dysfunction. This reference burst 8 years from 2004 to 2011. The reference burst from 2004 to 2008 published by Frustaci et al. (51) reported myocardial cell death in human diabetes through clinical research. Frustaci et al. (51) found that Angiotensin II (Ang II) might enhance oxidative damage, and activate cardiac cell apoptosis and necrosis *via* ventricular myocardial biopsies. This section mainly discussed the representative reference with the strongest citation burst duration from 2004 to 2011. We could conclude that the research hotspot of DCM was the myocardial cell death and PPAR pathway.

In addition, Table 5 showed the nearest burst duration was from 2019 to 2021 which represented the emerging trends of DCM. The first was published by Jia et al. (32), with a higher burst of 67.07. They constructed an update of mechanisms contributing to the clinical practice. Exosomes and miRNA might be emerging research directions in DCM mellitus. Methods such as β-type natriuretic peptide (BNP), exercise stress testing, and echocardiography did not appear to be accurate enough to screen for DCM. Besides, targeting SGLT2 might be a new treatment for DCM. Secondly, Jia et al. (32) published in Diabetologia with a burst of 35.09. Jia et al. (32) reported hyperglycemia and insulin resistance, increased AGEs, cardiac lip toxicity, activation of RAAS, coronary endothelial dysfunction, and exosome dysfunction, leading to mitochondrial dysfunction, oxidative stress, ER stress, and impaired calcium homeostasis. These pathophysiological abnormalities were associated with cardiac hypertrophy, fibrosis, death, stiffness, diastolic dysfunction, and heart failure. Aneja et al. (41) with a burst of 36.02 had the strongest citation burst duration from 2010 to 2016. This article provided insights into the pathogenesis, diagnostic challenges, and treatment options of DCM. It confirmed that high glucose played an important role in the pathogenesis of DCM, described the application of echocardiography or cardiac magnetic resonance in the auxiliary diagnosis of DCM, and summarized the current emerging therapies for DCM.

## Conclusions

The co-citation analysis and visualized network of the references about DCM were conducted by CiteSpace. Our study enabled scholars to intuitively display the basic knowledge structure and evolution process in the research field of DCM, and detected relevant hotspots and research trends. Research hotspots in this field mainly focused on the mechanism of metabolic disorder and imbalance caused by oxidative stress and inflammation in DCM under the background of genetic factors and were closely related to clinical manifestations such as heart failure, myocardial fibrosis, and cardiac hypertrophy. The research keywords mainly included “stress state,” “energy metabolism,” “autophagy,” “apoptosis,” “inflammation,” “myocardial fibrosis,” “PPAR,” etc. However, DCM still faces some urgent issues to be solved. First, there is a lack of a consistent definition of diabetic cardiomyopathy. Second, there is no consensus on the pathophysiological mechanisms. In addition, there is a lack of clinical diagnosis and treatment for this disease. All of the above have limited the understanding of DCM. Our study indicates that the cooperation between personnel needs to be strengthened, and the diagnosis, pathophysiological mechanisms, and treatment of DCM are the future research trends.

## Strengths and limitations

Through the application of bibliometrics and visual analysis, this paper presented a quantitative scientometrics method to provide doctors and researchers with trending frontiers in the field of DCM. The results will help clinicians and basic researchers intuitively understand patterns and trends in recognition. However, there were some limitations in this paper. Firstly, our paper only included research and reviews, and all were written in English. Therefore, it could not fully reflect the global situation in this field. Secondly, database variation was the limitation of bibliometric analysis. Due to the limitations of the software, only The WoS database was analyzed. Relevant software and analytical techniques can be developed to expand the scope of inclusion and obtain more comprehensive research literature in this field in the future.

## Data availability statement

The original contributions presented in the study are included in the article/supplementary material, further inquiries can be directed to the corresponding author/s.

## Author contributions

ST and DY conceived and designed the study. ST, DY, ZW, and LH conducted the statistical analyses. ST, DY, LZ, and LY drafted the manuscript. LL, JZ, and RY consulted and supplemented the relevant information. MS had full access to all the data and took responsibility for the integrity of the data and the accuracy of the data analysis. All authors participated in the interpretation of the results, revised the manuscript, read, and approved the final manuscript.

## Funding

This study was supported by grants from the National Natural Science Foundation of China (Grant No. 2017-GZR-011).

## Conflict of interest

The authors declare that the research was conducted in the absence of any commercial or financial relationships that could be construed as a potential conflict of interest.

## Publisher's note

All claims expressed in this article are solely those of the authors and do not necessarily represent those of their affiliated organizations, or those of the publisher, the editors and the reviewers. Any product that may be evaluated in this article, or claim that may be made by its manufacturer, is not guaranteed or endorsed by the publisher.
